# Association between Clinical and Doppler Echocardiographic Parameters with Sudden Death in Hemodialysis Patients

**DOI:** 10.5935/abc.20160098

**Published:** 2016-08

**Authors:** Silvio Henrique Barberato, Sérgio Gardano Elias Bucharles, Marcia Ferreira Alves Barberato, Roberto Pecoits-Filho

**Affiliations:** 1 Cardioeco - Centro de Diagnóstico Cardiovascular, Curitiba, PR - Brazil; 2 Fundação Pró Renal, Curitiba, PR - Brazil; 3 Pontifícia Universidade Católica do Paraná, Curitiba, PR- Brazil

**Keywords:** Death Sudden, Cardiac, Renal Dialysis, Echocardiography, Doppler, Hypertrophy, Left Ventricular, Risk Factors

## Abstract

**Background:**

Sudden cardiac death (SCD) is the leading cause of death in maintenance hemodialysis (HD) patients, but there is little information about underlying risk factors.

**Objectives:**

Evaluate the association between clinical and echocardiographic variables with SCD on HD patients.

**Methods:**

Retrospective nested case-control study on chronic HD patients who were prospectively followed. The primary endpoint was SCD. Variables were compared by Student t test, Mann-Whitney or Chi-Square, and independent predictors of SCD were evidenced by multivariate logistic regression.

**Results:**

We followed 153 patients (50 ± 15 years, 58% men) for 23 ± 14 months and observed 35 deaths, 17 of which were SCD events. When compared to the control group (matched for gender, age, and body mass index) there were no differences regarding time on dialysis, traditional biochemical parameters, blood pressure, smoking, use of cardiovascular protective drugs, ejection fraction, left ventricular dimensions, and diastolic function indices. On the other hand, in the SCD group, we found a higher prevalence of previous heart failure, acute myocardial infarction and diabetes, greater left ventricular mass index, greater left atrial size and lower global myocardial performance. After multivariate logistic regression analysis, diabetes (OR = 2.6; CI = 1.3-7.5; p = 0.023) and left ventricular mass index ≥ 101 g/m^2.7^ (OR = 1.04; CI = 1.01-1.08; p = 0.028) showed independent association with SCD events.

**Conclusions:**

HD patients with diabetes mellitus and left ventricular hypertrophy appear to have the highest risk of SCD. Preventive and therapeutic strategies should be encouraged in addressing these risk factors to minimize the occurrence of SCD in HD patients.

## Introduction

Cardiovascular diseases are the main cause of morbidity and mortality in patients with chronic kidney disease (CKD) in its more advanced stages, especially in patients undergoing dialysis.^1^ Sudden cardiac death (SCD) is the most common cause of death in individuals undergoing maintenance hemodialysis (HD) - it occurs 30 times more than in the general population and is responsible for up to 25% of deaths in this group of patients.^2^ SCD is characterized as unexpected death of cardiac origin that occurs within the first hour of the onset of symptoms in a patient that does not present with a known potentially fatal cardiac condition.^3^ Among documented cases of cardiac arrest in patients under HD, the main cause is ventricular arrhythmia (fibrillation or tachycardia) and, even resisting the acute event, the percentage of survival in this group of individuals is approximately 15% at the end of one year.^4^ The high prevalence of obstructive coronary artery disease on HD does not fully explain the excessive risk of SCD given that other potential pathological precipitants seem to be involved.^5^ In this clinical context, the identification of risk factors associated with the occurrence of SCD in a population of HD patients in the "real world" may aid in the prognostic assessment and selection of intervention strategies. Although several variables have been linked to the occurrence of SCD in terminal stages of CKD,^6^ there is a lack of studies that simultaneously approach clinical and cardiac morphophysiological aspects. It is known that the discovery of Doppler echocardiographic alterations in the left ventricle (LV), such as hypertrophy, dilatation, systolic dysfunction and diastolic dysfunction, is an important step to characterize individuals with higher risk.^7^ It is believed that cardiac structural abnormalities, added to the regular stress of traditional HD sessions (electrolyte and blood volume changes), may trigger fatal cardiac arrhythmias.^6,8^ The objective of this study is to evaluate the association between clinical and Doppler echocardiographic parameters and SCD occurrence in stable patients undergoing HD.

## Methods

### Population

Retrospective case-control nested study on a cohort of HD patients, with parameters prospectively collected in two renal replacement therapy centers. Inclusion criteria were as follows: age ≥ 18 years; maintenance HD therapy (time ≥ 3 months, definitive vascular access, and four hour sessions, three times a week); and signed consent form. Exclusion criteria were: recent hospital admission (< 30 days); malignancies; active infection; non sinus rhythm; significant valvular heart disease (any valvular stenosis ≥ moderate; valve prosthesis); and pericardial effusion. All patients underwent HD with standard dialysate (3.0 meg/L calcium concentration and 2.0 meg/L potassium concentration), through equipment with polysulfone dialyzers regulated with minimal blood flow of 350 ml/minute and dialysate flow of 500 ml/minute. The estimate of dry weight (volume to be removed by ultrafiltration in each HD session) was done by clinical criteria of hydration, blood pressure behavior during the session, and electrical bioimpedance (when applicable), as determined by the assisting doctor in the HD room.^9^ Body surface was calculated according to Dubois & DuBois equation (0.20247 x weight^0.425^ x height^0.725^). Body mass index (BMI) was calculated through the division of weight (kg) by the square of the height (m). Blood pressure, heart rate, weight, and height were measured at the time of the exam. The ethics committee for research of the institution approved the study protocol in accordance to resolution 196/96 of the National Health Council, and informed consent was obtained from all patients.

### Clinical Data and Outcome

Demographics, traditional cardiovascular risk factors (diabetes mellitus, arterial hypertension, dyslipidemia, smoking), previous cardiovascular diseases (congestive heart failure, acute myocardial infarction, stroke or peripheral arterial disease), intradialytic hypotension (2 or more episodes of symptomatic hypotension requiring HD interruption for ≤ 6 months),^10^ use of cardiovascular medication and lab findings were obtained through record analyses. Cause of death was obtained through the analysis of death certificates as well as interviews with the responsible assisting physician when necessary. The outcome of the study was SCD, defined as unexpected death, less than 1 hour after appearance of symptoms. Other events, such as non-sudden cardiovascular death and non-cardiovascular death, were excluded from the analysis.

### Doppler echocardiogram

The exams were performed on an interdialytic day, between 12 and 6 pm, except for Mondays, with the objective of minimizing the influence of afterload influence on the various Doppler echocardiographic indices.^7^ All patients were examined with HD echocardiograph 7XE (Phillips Inc., Bothell, Washington, EUA) equipped with 2.5 MHz transducer, for the complete M-mode, bidimensional and Doppler (pulsatile, continuous, colored and tissue) studies. The following parameters were obtained by the M-mode: anteroposterior diameter of the left atrium; diastolic interventricular septum thickness; diastolic thickness of the lower lateral wall; end-diastolic and end systolic LV diameter. Left ventricular mass was estimated by the Devereux formula and indexed in two ways: by body surface in square meters and by the height to the power of 2.7.^11^ Left ventricular hypertrophy was defined by the presence of mass/height^2.7^ ≥ 45 g/m^2.7^ for women, and ≥ 49 g/m^2.7^ for men.^11^ Systolic function was assessed by calculating the ejection fraction through Simpson's method. Mitral transvalvular flow was recorded at apical 4-chamber view with a sample of the pulsatile Dopplers positioned between the extremities of the cusps of the mitral valve, measuring the velocities of early rapid filling (E wave), atrial contraction (A wave), E/A ratio, and deceleration time of the E wave (DT). Myocardial performance index (MPI, or Tei index), representing the global myocardial performance, was calculated by the equation a-b/b, where a = intermitral interval (time between the end of a mitral flow and the beginning of the next one); and b = aortic flow ejection time, obtained from LV outflow.^12^ Myocardial tissue Doppler velocities were recorded at apical 4-chamber view with a sample consecutively positioned in the junction of the lateral and septal walls of the LV and the mitral annulus. Early diastolic velocity (e'), diastolic velocity of atrial or late contraction (a'), systolic velocity (s') of the annulus were measured, and the ratios e'/a' and E/e' (average of both sides of the mitral annulus) were calculated.^13^ Left atrium volume was determined by the bidimensional through biplanar Simpson's and indexed in two ways: by body surface in square meters, and by the height to the power of 2.7.^14^

### Statistical Analysis

The group that suffered SCD was retrospectively identified within the study population and matched for gender, age and BMI in the proportion: 1 case: 2 controls. The results were expressed as means and standard deviation (for continuous variables with parametric distribution), median (for continuous variables with non-parametric distributions) and percentage (for categorical variables). The groups were compared by unpaired Student's t test, Mann-Whitney or chi-squared test. Independent association between the various studied parameters and the occurrence of the outcome was tested by the multivariate conditional logistic analysis to derive odds ratios with 95% confidence interval. The significant univariate predictors were added to the multivariate model (entry and retention with significance of 0.1 and 0.05, respectively). For the definition of the partition value, we used a receiver-operator curve analysis (ROC). The level of statistical significance was defined as p < 0.05. The statistical program "SPSS 13.0 for Windows" (SPSS Inc., Chicago, IL USA) was used for all analyses.

## Results

### Basic characteristics of the general population

Basic demographic, clinical, biochemical and Doppler echocardiograph characteristics of the individuals forming the study's general population are depicted in Table 1. The study followed 153 HD patients, with a mean age of 50 ± 15 years, 89 men (58%), and 64 women. At the beginning of the study, 45% presented with arterial hypertension, 23% had diabetes mellitus, 25% had dyslipidemia, 11% were smokers, and 9% were overweight. Previous history of heart failure was present in 31%, acute myocardial infarction in 5%, and stroke in approximately 3%. Most patients (80%) were on antihypertensive medication, especially converting enzyme inhibitors (47%), beta-blockers (16%), alpha-blockers (13%), calcium channel antagonists (12%), and angiotensin receptor blockers (12%), isolated or together. LV dilatation was present in 26% of patients, LV hypertrophy in 89%, systolic dysfunction in 18%, and diastolic dysfunction in 73% - these echocardiographic findings were either isolated or in association.

**Table 1:** Main clinical characteristics and basal cardiovascular riskfactors in the general study population

	**Total (n = 153)**
Age (years)	50 ± 15
Following (months)	23 ± 14
Males (%)	58
Time on HD (months)	22*
Arterial hypertension (%)	45
Diabetes mellitus (%)	23
Dyslipidemia (%)	25
Smoking (%)	11
Left ventricular hypertrophy (%)	89
Systolic dysfunction (%)	18
Diastolic dysfunction (%)	73
Previous AMI (%)	5
Previous HF (%)	31
Previous stroke (%)	3

Data presented as mean ± SD, median

* or percentages. HD: hemodialysis; AMI: acute myocardial infarction; HF: heart failure.

### Outcomes

There were 35 deaths during the 23 ± 14 month period of the study. Among the events, there were 8 deaths of non-cardiovascular etiology, 10 non-sudden cardiovascular deaths, and 17 sudden cardiac deaths in the general study population. This group of 17 patients, characterized as "cases", was matched by gender, age, and BMI, with 34 controls who were alive when their respective "case" died.

#### Differences in patients with SD and controls

Table 2 depicts the main clinical and biochemical differences between individuals who suffered SCD and those who did not. There were no significant differences in age, gender, BMI, period under HD, blood pressure and lab parameters such as hemoglobin, albumin, and calcium-phosphorus product. In addition, there was presence of similar proportions of history of arterial hypertension, smoking, and intradialytic hypotension, as well as the use of beta-blockers or angiotensin converting enzyme inhibitors. Patients who had SCD presented a higher prevalence of heart failure, previous myocardial infarction, and diabetes mellitus. Table 3 shows a comparison of the main Doppler echocardiograph characteristics of SCD patients and controls. Similarities were found in LV dimensions, ejection fraction, and diastolic function parameters. On the other hand, SCD patients presented a larger left ventricular mass (indexed by both methods), lower global myocardial performance (assessed by MPI) and larger left atrium (assessed by the indexed volume divided by the height to the power of 2.7).

**Table 2:** Comparison of clinical and biochemical characteristics between patients with sudden cardiac death and the control group

**Variable**	**Sudden cardiac death (n = 17)**	**Control (n = 34)**	**p value**
Age (years)	56 ± 16	52 ± 13	0.43
Male (%)	70	62	0.75
BS (m^2^)	1.69 ± 0.2	1.73 ± 0.2	0.23
BMI	25 ± 6	25 ± 5	0.68
Time on HD (months)	26	22	0.60*
Systolic HR (mmHg)	153 ± 23	141 ± 27	0.37
Diastolic HR (mmHg)	87 ± 11	84 ± 11	0.54
Arterial hypertension (%)	70	59	0.54
Diabetes mellitus (%)	53	21	0.03
Use of beta-blockers (%)	12	26	0.30
Use of IACE (%)	59	47	0.55
Smoking (%)	6	6	1.00
Previous HF (%)	59	23	0.03
Previous AMI (%)	12	0	0.04
Hypotension ID (%)	12	6	0.30
Hemoglobin (g/dl)	10.1 ± 2.4	10.0 ± 2	0.27
Albumin (mg/L)	3.4 ± 0.4	3.7 ± 0.6	0.48
Ca x P Product	49 ± 16	53 ± 18	0.50

BS: body surface; BMI: body mass index; HD: hemodialysis; HR: heart rate; IACE: inhibitor of angiotensin converting enzyme; HF: heart failure; AMI: acute myocardial infarction; ID: intradialytic; Ca x P: calcium x phosphorus.

* -Mann-Whitney test.

**Table 3:** Comparison between Doppler echocardiographic characteristics between patients with sudden cardiac death and the control group

**Variable**	**Sudden cardiac death (n = 17)**	**Control (n = 34)**	**p value**
LVEDD (mm)	53 ± 7	51 ± 6	0.52
LVESD (mm)	36 ± 6	35 ± 7	0.62
LVMI (g/m^2^)	211 ± 66	171 ± 45	0.014
LVMI (g/m^2.7^)	121 ± 43	92 ± 24	0.003
EF percentage	67 ± 8	67 ± 9	0.76
E (cm/s)	89 ± 27	77 ± 23	0.12
A (cm/s)	94 ± 31	83 ± 29	0.22
E/A	1.2 ± 0.9	1.1 ± 0.7	0.66
IVRT (ms)	117 ± 31	103 ± 28	0.13
DT (ms)	174 ± 51	198 ± 57	0.15
MPI	0.64 ± 0.1	0.53 ± 0.1	0.019
DLA (mm)	38 ± 5	37 ± 4	0.53
LAVI (ml/m2)	41 ± 17	34 ± 11	0.13
LAVI (ml/m alt ^2.7^)	12 ± 5.5	9 ± 3.3	0.032
e’ (cm/s)	7.1 ± 1	7.3 ± 1.8	0.57
a’ (cm/s)	7.8 ± 2.8	8.2 ± 2.5	0.37
s’ (cm/s)	10 ± 2.9	11 ± 3.1	0.58
e’/ a’	0.71 ± 0.2	0.68 ± 0.2	0.53
E/e’ media	13 ± 5	11 ± 5	0.30
Valvular calcification (%)	53	29	0.13

LVEDD: left ventricular end-diastolic diameter (LV); LVESD: LV end-systolic diameter; LVMI: left ventricular mass index; EF: ejection fraction; E: early rapid filling velocity; A: atrial contraction velocity; IVRT: isovolumetric relaxation time; DT: deceleration time; MPI: myocardial performance index; DLA: anteroposterior diameter of the left atrium; LAVI: left atrial volume index; e’: early diastolic velocity of the mitral annulus; a’: late diastolic velocity of the mitral annulus; s’: systolic velocity of the mitral annulus.

### Independent Association to SD

Significant variables in the univariate analysis were included in the multivariate logistic regression model, corrected by gender, age, and BMI. To avoid statistical collinearity and diminish the influence of weight/volume, we decided to include the indexed left ventricular mass divided by the height to the power of 2.7, instead of the indexed mass divided by body surface. There was no independent association between the occurrence of SCD and history of heart failure or myocardial infarction, left atrium size and global myocardial performance by MPI (Table 4). On the other hand, history of diabetes mellitus (OR = 2.6; CI = 1.3-7.5; p = 0.023), and indexed left ventricular mass (RC = 1.04; CI = 1.01-1.08; p = 0.028) appeared as parameters independently associated to SCD. In particular, individuals with left ventricular mass ≥ 101 g/m^2.7^ (ROC curve, p = 0.02) showed higher risk of the outcome.

**Table 4:** Multivariate logistic regression analysis of the association between clinical and Doppler echocardiographic parameters with sudden death

	**Sudden cardiac death**		
	**OR**	**CI 95%**	**p value**
Diabetes	2.6	1.3-7.5	0.023
HF			ns
AMI			ns
MPI			ns
LAVI (ml/m alt^2.7^)			ns
LVMI (por g/m^2.7^)	1.04	1.01-1.08	0.028

OR: odds ratio; CI 95%: confidence interval; HF: heart failure; AMI: acute myocardial infarction; MPI: myocardial performance index; LAVI: left atrial volume index; LVMI: left ventricular mass index.

## Discussion

The main finding of this study was to point out the independent association between the presence of diabetes mellitus and left ventricular hypertrophy with higher risk of SCD in patients on HD. SCD is the main cause of death in patients under chronic dialytic treatment.^2^ Recent estimates show that approximately 25% of deaths in patients on dialysis is due to SCD, especially after severe arrhythmias and/or unexpected heart arrest.^2^ Despite the high incidence of the phenomenon, understanding of risk factors and underlying pathophysiological mechanisms remains limited, which restricts the outlining of preventive therapeutic strategies. It is noteworthy that the incidence of SCD is similar in prospective studies with populations of patients on HD^15^ or peritoneal dialysis.^16^ Pathophysiology of SCD is complex and uncertain, but it is believed that there needs to be an interaction between a transitory event (sudden change in volume and/or electrolyte concentration, as a trigger) and a pathological substrate (such as "uremic cardiomyopathy"). The combination of these alterations would be responsible for triggering complex arrhythmias and hemodynamic instability, followed by circulatory collapse.^8^

CKD induces several structural modifications in the cardiovascular system, the most frequent of which is left ventricular hypertrophy.^7^ The pathophysiology of LV hypertrophy in CKD is multifactor and depends on the interaction of numerous factors, such as preload increase (by volume overload, anemia, and high flows in the arteriovenous fistula), afterload increase (arterial hypertension and vascular calcification), and other particular consequences of uremia (oxidative stress, systemic inflammation, secondary hyperparathyroidism, vitamin D deficiency, and hyperphosphatemia.^1,17^ Myocardial hypertrophy brought on by the uremic environment shows distinct characteristics, especially intermyocardial fibrosis and arteriolar thickening, which promote a decrease of ventricular compliance and electrical instability, predisposing the development of advanced diastolic dysfunction and potentially fatal arrhythmias.^13,17^ Our findings corroborate the notion that left ventricular mass, known predictor of general mortality and cardiovascular events,^18^ is also linked to the specific occurrence of SCD in HD patients. In parallel, it has been shown that the increase of left ventricular hypertrophy throughout time during dialysis is related to a higher chance of SCD.^19^

On the other hand, several Doppler echocardiographic variables, indicative of cardiovascular risk in different clinical contexts, do not show independent association to the risk of SCD in the HD population. Left ventricular systolic dysfunction, represented by a reduced ejection fraction, is a classic predictor of SCD in patients with heart failure, dilated cardiomyopathy, and myocardial infarction.^20^ Contrastingly, a reduced ejection fraction does not necessarily operate as an independent risk factor for SCD in HD patients, as evidenced in literature^21^ and in the present study. The same behavior has been observed with other Doppler echocardiographic markers of cardiovascular risk, such as MPI and left atrium volume, which did not reach statistical significance in the multivariate analysis. Importantly, a previous study suggested that pro-BNP serum biomarkers and troponin are able to substitute the importance of Doppler echocardiographic data in the prediction of SCD.^16^ However, the analysed population differed from that of the present study because it constituted exclusively of patients on peritoneal dialysis and with smaller mean left ventricular mass.^16^ The dosage of such biomarkers was not done in our sample.

Among clinical parameters, only diabetes mellitus was independently associated to the SCD phenomenon, after correction by gender, age, and BMI. Diabetes mellitus is, in medical literature, a known independent risk factor for SCD. Several pathophysiological mechanisms for the genesis of SCD in diabetic patients have been suggested, such as long QT interval secondary to nocturnal hypoglycemia (proarrhythmic environment),^22^ obstructive coronary artery disease,^23^ and autonomic neuropathy.^24^ Additionally, there seems to be a parallelism between inadequate glycemic control and increased risk of SCD. Diabetics on HD with glycated hemoglobin ≥ 8% present increased risk of SCD when compared to patients with tight glycemic control (< 6%).^25^

Obstructive coronary artery disease, although frequently observed in patients with advanced CKD, does not seem to have a clear association with the occurrence of SCD in patients on HD. A prospective study evaluating risk factors for SCD in an HD cohort did not point to coronary artery disease as a variable that was significantly associated to the outcome.^21^ Accordingly, a study investigating the cause of SCD through autopsies in patients who were on HD found acute myocardial infarction in only 5.7% of cases.^26^ In the present study, we did not exclude the presence of obstructive coronary artery disease through functional or anatomic tests; however, history of myocardial infarction did not have independent prognostic significance for an outcome of SCD, as observed in a previous report.^21^

It is known that hyperkalemia may determine a higher risk of ventricular arrhythmias, and a pre-dialysis potassium serum level > 6.0 meq/L is associated to a higher risk of SCD.^21^ One of the limitations of our study, during the research observation period, is that it was not possible to retrieve potassium serum levels from all patients. However, the group of patients received dialysis treatment with potassium concentration in the dialysate of 2.0 meq/L, which offers greater protection against serum variations when compared to potassium concentrations in the dialysate of under 2.0 meq/L.^27^ Finally, bone and mineral metabolism disorders, often observed in HD patients,^28^ may be related to the phenomenon of SCD. It has been shown that patients with hyperphosphatemia on HD seem to be more at cardiovascular risk and risk of SCD than patients with normal parathormone and phosphorus levels.^29^ However, in our analysis, calcium and phosphorus levels were similar among those who suffered SCD and those in the control group. Our study has, besides the aforementioned, other limitations: retrospective design, relative low number of events (except for the multivariate analysis), rigour of exclusion criteria, and the absence of the dosage of serum biomarkers (such as proBNP and troponin).

## Conclusions

We concluded that, in clinically stable patients, the occurrence of SCD, during regular maintenance HD treatment, was independently associated to the history of diabetes mellitus and to the left ventricular mass. Preventive and therapeutic strategies must be encouraged in the approach of these risk factors to decrease the occurrence of SCD. It will be necessary to have new, more comprehensive, and more prospective studies, capable of corroborating our findings, showing other variables that are potentially related to SCD in CKD environment and investigating the adoption of interventions that minimize the risk of the event in this group of individuals.
